# The Effect of Crude Oil Stripped by Surfactant Action and Fluid Free Motion Characteristics in Porous Medium

**DOI:** 10.3390/molecules29020288

**Published:** 2024-01-05

**Authors:** Qingchao Cheng, Guangsheng Cao, Yujie Bai, Ying Liu

**Affiliations:** Key Laboratory of Enhanced Oil & Gas Recovery of Ministry of Education, Northeast Petroleum University, Daqing 163318, China; nepu_cheng@163.com (Q.C.); a1416117553@163.com (Y.L.)

**Keywords:** porous medium, oil stripping efficiency, motion characteristics, interfacial parameters

## Abstract

The surfactant solution is crucial in facilitating the spontaneous imbibition process for the recovery of oil in tight reservoirs. Further investigation is required to examine the fluid flow in porous mediums and the process of crude oil stripping by a surfactant solution during spontaneous imbibition. Hence, this study aims to determine the free motion properties of oil and water in porous mediums using the finite-element approach to solve the multiphase flow differential equation, taking into account the capillary pressure. An investigation was conducted to examine the impact of oil viscosity and interfacial tension on the mean liquid flow rate and oil volume fraction. An experimental study was conducted to investigate the impact of surface tension, interfacial tension, and wetting angle on crude-oil-stripping efficiency. The findings indicate that the stripped crude oil migrated through porous mediums as individual oil droplets, exhibiting a degree of stochasticity in its motion. When the interfacial tension is reduced, the average velocity of the fluid in the system decreases. The crude oil exhibited a low viscosity, high flow capacity, and a high average flow rate within the system. Once the concentration of the surfactant solution surpasses a specific threshold, it binds with the oil to create colloidal aggregates, resulting in the formation of micelles and influencing the efficiency of the stripping process. As the temperature rises, the oil-stripping efficiency also increases. Simultaneously, an optimal range of wetting angle, surface tension, and interfacial tension could enhance the effectiveness of removing oil using surfactant solutions. The research results of this paper enrich the enhanced oil recovery mechanism of surfactant and are of great significance to the development of tight reservoirs.

## 1. Introduction

Surfactants are well acknowledged as a crucial technique for enhancing the productivity of tight reservoirs [1,2,3]. Spontaneous imbibition, facilitated by surfactants, is a successful development technique for tight reservoirs due to their inherent low porosity and permeability [4,5,6]. The function of surfactants in imbibition can be classified into two distinct groups. One is a surfactant with the ability to achieve ultra-low interfacial tension. Its principal function is to facilitate imbibition recovery through processes such as emulsifying stripping and thermal diffusion. Furthermore, there is a surfactant that has the potential to significantly alter the wetting capacity of the rock surface. Crude oil flows out from the pores and accumulates on the rock surface as droplets [7]. Upon entering the pores of the reservoir, the surfactant solution has the ability to interact with the crude oil, resulting in the formation of an emulsion. Additionally, the surfactant solution serves the purpose of stripping the oil from the reservoir [8,9,10]. Several investigations have demonstrated the impact of surfactant solutions on imbibition [11,12,13,14,15]. The imbibition process of an oil–water mixture is significantly influenced by the interfacial tension and the density difference between the two phases [16]. Furthermore, the specific surfactant employed has a crucial role in determining the interfacial tension between oil and water, thereby impacting the recovery of oil through spontaneous imbibition [17]. The process of imbibition recovery is influenced by variations in reservoir physical property, rock wettability, fracture number, and crude oil viscosity [18,19]. Multiple techniques exist for investigating spontaneous imbibition. The core spontaneous imbibition experiment is a widely-used and traditional experimental method. The utilization of nuclear magnetic resonance technology allows for the determination of the variations in the composition of crude oil in tight core samples with distinct pore shapes [20]. The nanochannel multi-scale model has also been utilized for studying the imbibition process, where the spatial arrangement of pores and cracks also influences the duration of imbibition [21]. X-ray computed tomography experiments are employed to generate digital cores, which can be integrated with a lattice Boltzmann multiphase model to investigate the impact of varying hydrophilic strengths on the development of imbibition fronts in micropores and the recovery of non-wettable fluid during the spontaneous imbibition process of tight sandstone [22]. Imbibition mathematical models have also been utilized to represent imbibition recovery [23]. The effects of shape factor (SF), characteristic length (CL), and boundary conditions (BC) on the rate of self-absorption have been investigated to improve the simulation of self-absorption oil production in naturally fractured reservoirs using the derived shape factors [24]. The application of fractal theory has been utilized to characterize the intricate pore structure of sedimentary rocks and to simulate the movement of fluids in porous mediums. A fractal geometry model was employed to replicate the spontaneous imbibition of the wet phase in a porous medium [25]. In order to investigate spontaneous imbibition in porous mediums, dynamic pore network models are utilized. The practicality of this method has been generalized by using several types of ideal pore elements to depict complex pore spaces and by conducting numerous case studies to verify the accuracy of pore network models [26,27]. Nevertheless, the flow of the fluid has not been verified, necessitating additional research. This research investigates the free motion characteristics of crude oil in a porous medium within a tight reservoir, specifically under the condition of spontaneous imbibition. The study involves the development of a multiphase fluid flow equation and the use of the finite-element method. The experiment was conducted to assess how the type, concentration, and temperature of the surfactant impact the stripping of crude oil from the rock surface. An analysis was conducted on the wetting angle, interfacial tension, surface tension, and stripping efficiency of crude oil. The findings of this study offer technical assistance for the effective exploitation of tight oil reserves.

## 2. Results and Discussion

### 2.1. Motion Characteristics of Crude Oil in Porous Medium under Spontaneous Imbibition

#### 2.1.1. Effect of Interfacial Tension on Crude Oil Flow

**Figure 1:**
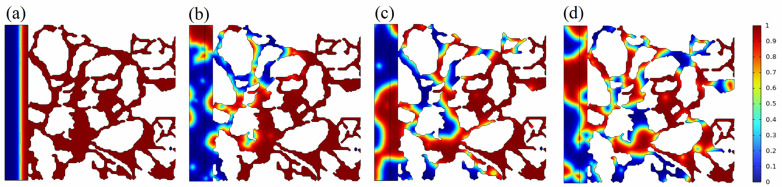
Oil–water distribution characteristics in porous medium at different time points. (**a**–**d**) are the characteristics of oil–water distribution at 0 ms, 20 ms, 40 ms, and 60 ms, respectively.

The dimensions of the porous medium model were 50 μm × 50 μm, with each individual pore having a radius of approximately 5 μm. The simulation was conducted over a time period of 200 ms. The density of the crude oil was 800 kg/m^3^, and its viscosity was 5 mPa·s. The density of the water was 1000 kg/m^3^, and its viscosity was 0.5 mPa·s. The interfacial tension between the oil and water was 0.01 mN/m. The calculation was performed using the COMSOL Multiphysics simulator. The distribution properties of the oil and water phases in the porous medium were estimated at different time intervals using the method. The distribution characteristics were computed according to the diagram depicted in Figure 1.

**Figure 2:**
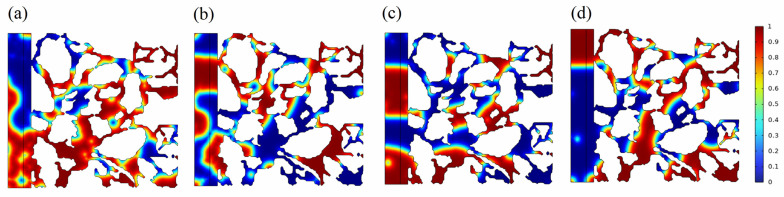
Characteristics of oil–water distribution in porous medium after 200 ms under different oil–water interfacial tension. The oil–water interfacial tensions for (**a**–**d**) are 0.001 mN/m, 0.005 mN/m, 0.01 mN/m, and 0.05 mN/m, respectively.

The red scale represents the variation in the saturation of the oil phase. When the oil phase saturation is zero, the surfactant solution saturation is one. Over time, the oil and water phases become more mobile, allowing the surfactant solution to penetrate the pores. Crude oil is progressively removed from the pore surface, and once removed, it eventually forms into oil droplets due to the oil–water interfacial tension. The crude oil, in the form of oil droplets, can then be more efficiently carried through the porous medium. This shows that crude oil stripping and emulsification have a significant impact on fluid flow inside the porous medium of a tight reservoir. The distribution of the oil phase within the pores under different oil–water interfacial tensions was further calculated, and the results are shown in Figure 2.

When the interfacial tension between the oil and water phases in the porous medium was 0.001 mN/m, the oil phase had a significant presence. However, the distribution of oil and water within the porous medium was intricate. Conversely, the oil phase exhibited a comparatively low saturation when the interfacial tensions were relatively high. This indicated that a decrease in interfacial tension improved the mixing of fluids in porous mediums, but it hindered oil-stripping efficiency. Hence, in order to effectively exploit tight reservoirs, it was crucial to regulate the oil–water interfacial tension within an appropriate range. This could ensure the efficient extraction of crude oil and also enhance its emulsification properties, facilitating its movement through the porous medium.

The concentration of surfactant also affected the interfacial tension between oil and water, while the magnitude of the interfacial tension had a large impact on the oil volume fraction and the average flow rate of the fluid. Therefore, it is necessary to further analyze the free flow characteristics of the fluid in the porous medium of tight rocks. This analysis should encompass the characteristics of change in oil volume fraction and average flow rate within the porous medium at various time intervals, while accounting for varied interfacial tensions.

**Figure 3:**
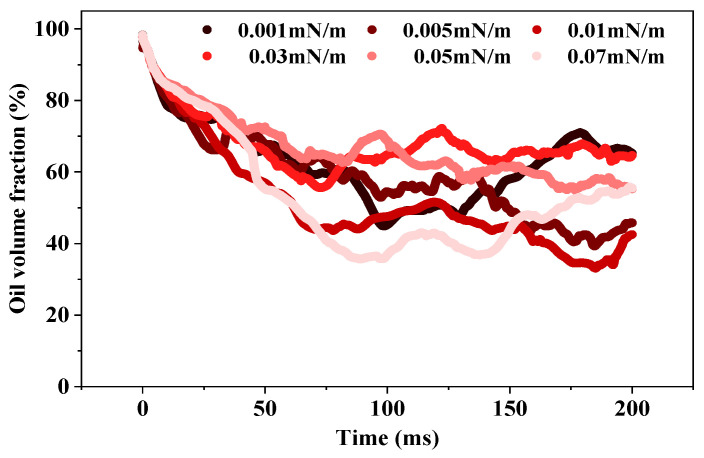
Variation in oil volume fraction with time at different interfacial tensions.

Figure 3 demonstrates that the oil volume fraction in the porous medium exhibited wave-like fluctuations under various interfacial tensions. This suggests that the movement of oil and water phases within the medium was somewhat random. At 200 ms, the oil volume fraction fell within the range of 50–70%. The occurrence of this scenario could be attributed to the re-entry of oil droplets into the porous medium from the aqueous phase during free motion, leading to a fluctuating reduction in the oil volume fraction within the porous medium.

**Figure 4:**
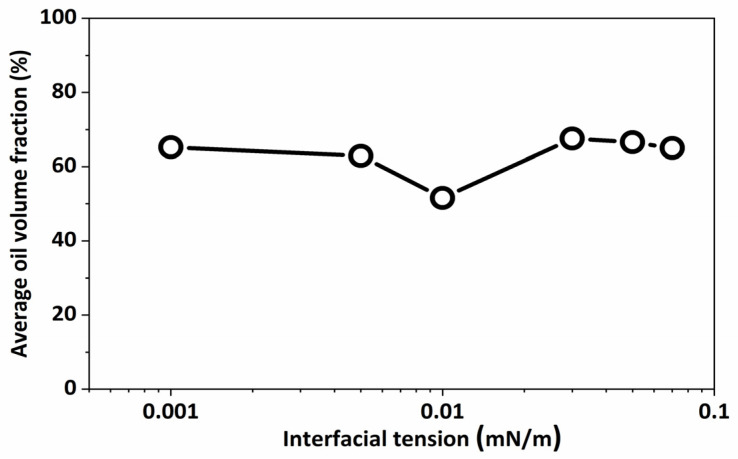
Variation in oil volume fraction in porous medium with different interfacial tensions at 200 ms.

Generally, the oil volume fraction in the porous medium dropped and subsequently increased as the interfacial tension increased. This suggests that extremely low interfacial tension could not improve the recovery of spontaneous imbibition (Figure 4). The interfacial tension must be regulated to provide optimal performance in both crude oil stripping and the formation of smaller emulsions for transportation through the porous medium.

**Figure 5:**
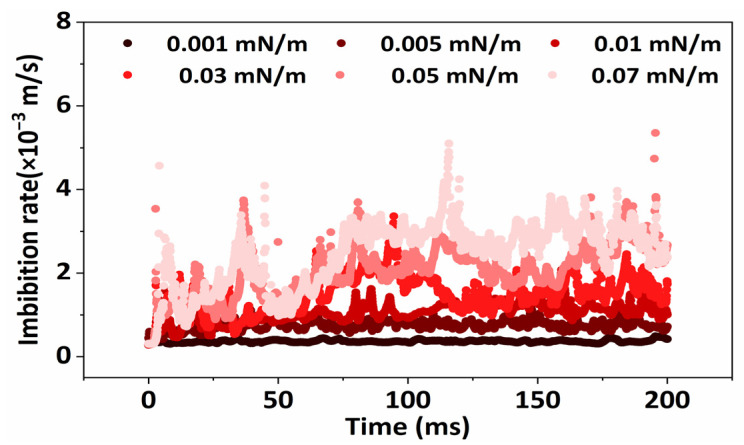
The velocity of spontaneous imbibition in porous medium under different interfacial tensions at different times.

As can be seen in Figure 5, a dynamic and stable change in the flow rate of the fluid within the porous medium was observed when the interfacial tension was below 0.01 mN/m. This means that the oil–water distribution in the fluid in the porous medium could not change how freely the fluid moves within the formation when the interfacial tension was in this range. Furthermore, in cases where the interfacial tension between water and oil exceeds 0.01 mN/m, the mean fluid flow rate undergoes a gradual alteration as the simulation time progresses. The distributions of oil and water within the porous medium have an impact on this variation. Moreover, as the crude oil is extracted from the rock surface, it transforms into an emulsion. The capillary force imparts energy to the emulsion particles, resulting in an increase in the overall average flow rate due to their accelerated movement within the porous medium.

**Figure 6:**
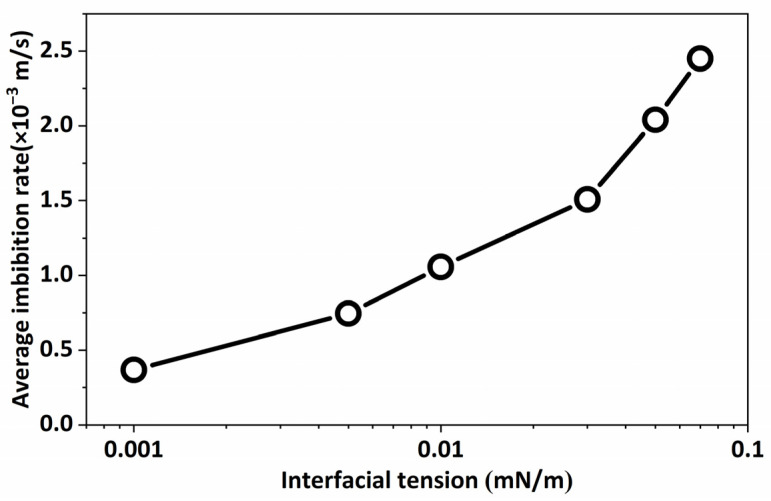
The average velocity of spontaneous imbibition in porous medium under different interfacial tensions.

Figure 6 illustrates that within the range of 0.001 mN/m to 0.07 mN/m, there was a correlation between interfacial tension and fluid flow rate. Specifically, when the interfacial tension was low, the average flow rate of the fluid in the system was also low. Conversely, as the interfacial tension between oil and water increased, the average flow rate of the fluid in the system demonstrated an upward trend. Nevertheless, this fluctuation in flow rate was characterized by a non-unidirectional flow; hence, the augmentation in flow rate does not inherently improve the retrieval of crude oil.

#### 2.1.2. Effect of Viscosity on Crude Oil Flow

The viscosity of the aqueous phase in the porous medium was set to be 0.5 mPa·s, and the oil–water interfacial tension was 0.02 mN/m. The oil phase viscosity was 1 mPa·s, 5 mPa·s, 10 mPa·s, 15 mPa·s, and 20 mPa·s, respectively. 

**Figure 7:**
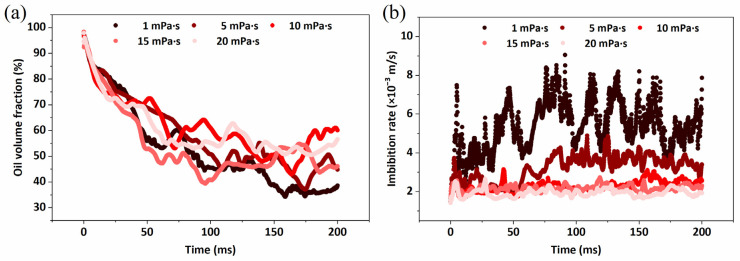
Motion characteristics of crude oil with different viscosities. (**a**) is the variation in the oil volume fraction by spontaneous imbibition with time; (**b**) is the velocity of spontaneous imbibition in the porous medium.

The changes in oil volume fraction and spontaneous imbibition velocity in the system at different moments are shown in Figure 7, oil with a low viscosity in porous medium flows more easily, discharges a larger volume of oil, and has a faster spontaneous imbibition rate. Hence, reducing the viscosity of crude oil through the injection of a suitable medium is a crucial method for enhancing imbibition recovery.

### 2.2. Factors Affecting Stripping Efficiency of Crude Oil

#### 2.2.1. Effect of Surfactant Types on Oil-Stripping Efficiency

The oil-stripping experiment serves as a direct indicator of the efficacy of various surfactant solutions in removing crude oil from rock particles. This is achieved by evaluating the performance of surfactants in the experiment, ultimately identifying the most suited surfactants. The analysis focused on the relationship between surface tension, interfacial tension, wetting angle, and other parameters to identify the elements that influence the removal of oil by surfactants.

**Figure 8:**
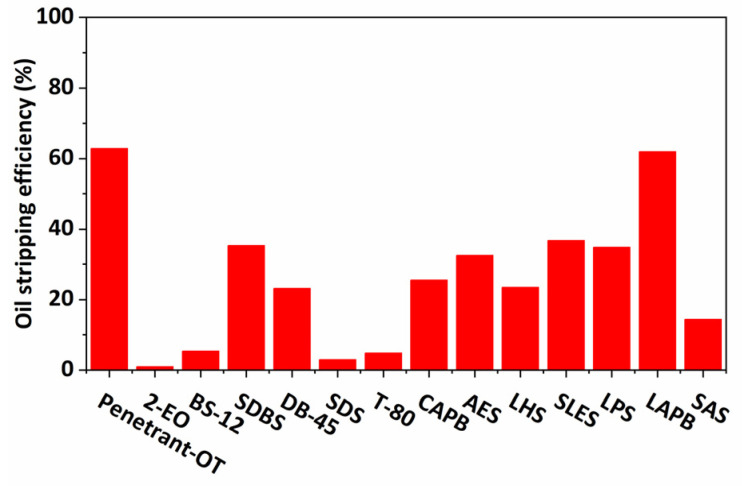
Oil-stripping efficiency of different surfactants (0.2% surfactant solution concentration).

It can be seen from Figure 8 that the oil-stripping efficiency of different surfactants varied widely when the surfactant concentration was certain. The oil-stripping efficiency of Penetrant-OT and LAPB was the most prominent, with 63% and 62%, respectively. Penetrant-OT and LAPB solutions were prepared at ratios of 0.1%, 0.4%, 0.6%, 0.8%, 1.0%, 1.2%, 1.4%, and 1.8% for the oil-stripping experiment.

**Figure 9:**
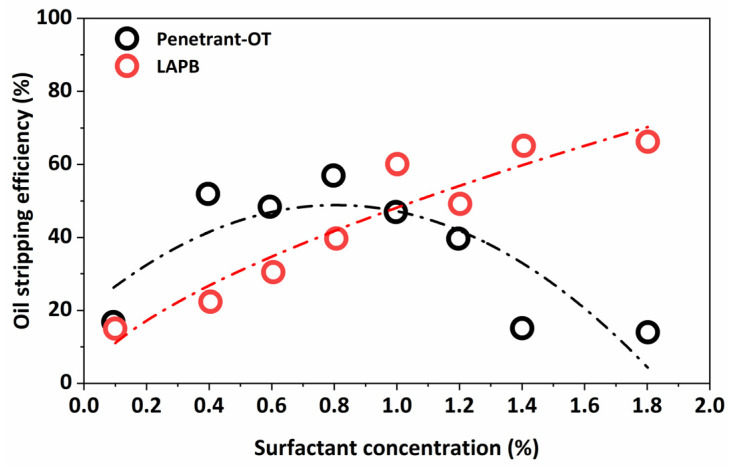
Oil-stripping efficiency under different surfactant concentrations.

Figure 9 demonstrates a positive association between the concentration and oil-stripping efficiency of LAPB. The stripping efficiency exhibited a consistent increase as long as the surfactant concentration of Penetrant-OT in the solution remained below 0.8%. When the concentration was higher than 0.8%, the oil-stripping efficiency of the solution began to decline. For a particular surfactant, when the concentration exceeded a certain concentration, it underwent a change in association from a single ion or molecule to a colloidal aggregation, that is, to form micelles, and the oil-stripping effect was affected. It is necessary to maintain a suitable concentration of surfactant during real production to prevent excessive concentration and its negative impact on the crude-oil-stripping action.

#### 2.2.2. Effect of Temperature on Oil-Stripping Efficiency

**Figure 10:**
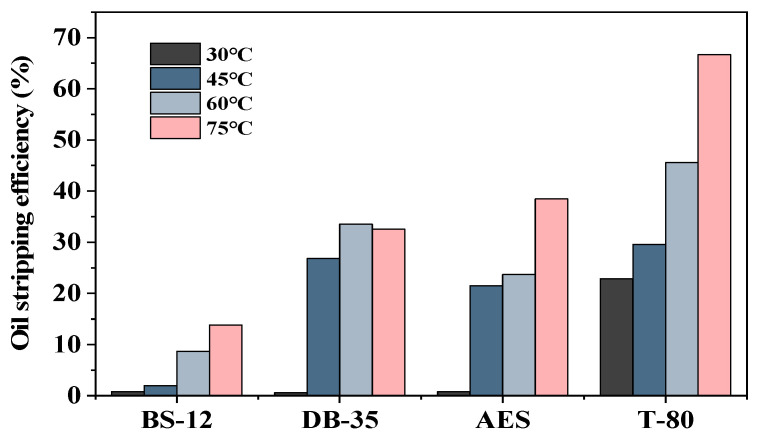
Oil stripping efficiency of surfactant solutions at different temperatures.

The BS-12, DB-35, T-80, and AES were considered. The further temperature changes in the experiment were 30 °C, 45 °C, 60 °C, and 75 °C, with the experimental findings presented in Figure 10.

It was observed that the oil-stripping efficiency rose as the temperature increased. This tendency was observed for all four surfactants. The stripping efficiency of the DB-35 surfactant reached a plateau when the temperature was above a particular threshold. The oil-stripping efficiency of T-80 consistently improved as the temperature increased. The surfactant exhibited more sensitivity to temperature due to the fact that T-80 readily forms a gel-like substance at low temperatures, limiting its ability to strip oil. However, it is only effective for stripping oil at high temperatures. The peeling of oil droplets is better achieved in elevated temperature settings.

#### 2.2.3. Effect of Fluid–Rock Interface Properties on Oil Stripping Efficiency

**Figure 11:**
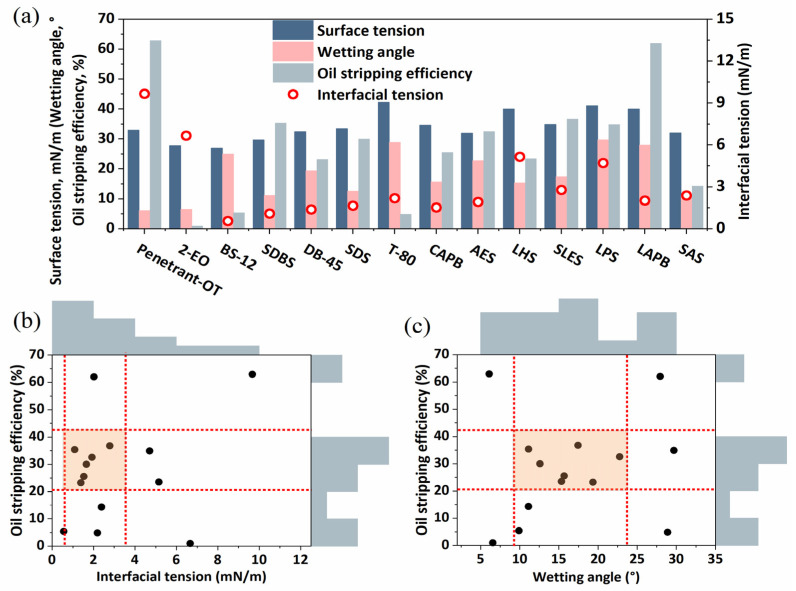
Oil-stripping efficiency and surfactant interfacial characteristics. (**a**) is the basic parameter of each type of surfactant solution; (**b**) is the relationship between the interfacial tension of the surfactant solution and oil-stripping efficiency; and (**c**) is the relationship between the wetting angle and oil-stripping efficiency.

Different surfactants were selected to analyze the relationship between surface tension, interfacial tension, wetting angle, and stripping efficiency, and the results are shown in Figure 11.

The results represent the corresponding surface tension, interfacial tension, and wetting angle (in contact with the glass sheet) for each type of surfactant solution. The measured surface tension values ranged from 25.4 mN/m to 41.2 mN/m, interfacial tensions ranged from 0.5 to 9.7 mN/m, and the wetting angles ranged from 6° to 29.7°, corresponding to oil-stripping efficiencies of 1% to 62.9%. Among the surfactant solution types tested thus far, there is not a linear relationship between interfacial tension, wetting angle, and oil-stripping efficiency, and an optimal interval exists for a high oil-stripping efficiency (Figure 11b,c), and higher oil-stripping efficiencies are obtained when the wetting angle of the rock ranges from 10° to 22°, and when the interfacial tension between oil and water is 2 mN/m or less. It indicates that under the interaction of oil, water, and rock, the existence of a reasonable range of wetting angle, surface tension, and interfacial tension makes it possible to enhance the stripping ability of crude oil.

The wetting phenomenon occurs when one fluid replaces another on a solid surface. The wetting degree determines whether the surface free energy (adhesion work) of the system can be lowered (raised) under constant temperature and constant pressure. The larger the reduction, the greater the degree of wetness. As a result, the relationship between the wetting degree and oil stripping efficiency must be clarified, as well as the interaction between the surface tension, wetting angle, and stripping efficiency.

Free energy (adhesion work) can be expressed by the T·Young equation.

(1) $$W_{a}=-\Delta G=\sigma_{g-l}(1+\cos\theta )$$

where −ΔG is the free energy; $W_{a}$ is adhesion work, mN/m; $\sigma_{g-l}$ is surface tension, mN/m; θ is wetting contact angle, °.

**Figure 12:**
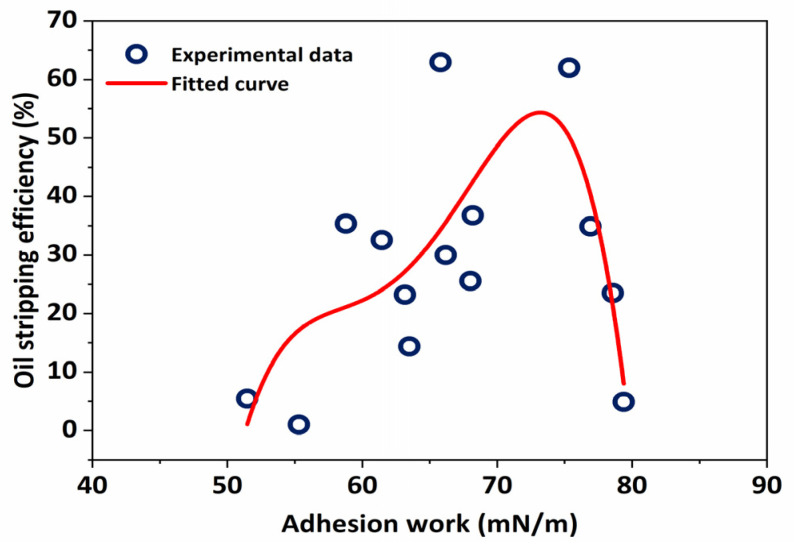
Relationship curve between adhesion work and oil-stripping efficiency.

The wetting phenomenon occurs when one fluid replaces another on a solid surface. It can be found that the adhesion work had a high oil-stripping efficiency within a certain range from Figure 12. When the adhesion work was 65–70 mN/m, the corresponding oil stripping efficiency could reach 46–70%. With the increase in adhesion work, the stripping efficiency increased first and then decreased. This is for the reason that high adhesion work results in most of the surfactant molecules being adsorbed on the surface of quartz particles, reducing the adsorption of other particles. There is a certain adsorption probability of crude oil adsorbed on the surface of oil sand particles, and most of the surfactant adsorbed on a single particle reduces the stripping efficiency of the whole system.

## 3. Theory and Experiment

### 3.1. Theory 

The free flow of crude oil and water within a porous medium can be considered a multiphase porous flow, where gravity, capillary forces, and interfacial tension between the oil and water phases play a role. The equation governing the flow of oil and water in a multiphase system is Equation (2).

(2) $$\frac{\partial \left(\rho_{m}A\right)}{\partial t}+\frac{\partial \left(GA\right)}{\partial z}=0$$

where $\rho_{m}$ is the fluid density, kg/m^3^; *A* is the pore cross-section area, m^2^; *G* is the gravitational density, N/m³; and *t* is the time, s.

Equation (3) expresses the flow of oil and water in a porous medium under gravity.

(3) $$\frac{\partial }{\partial t}\left(\rho_{m}w_{m}\right)+\frac{\partial }{\partial z}\left(\rho_{m}w_{m}^{2}\right)=-\frac{\partial p}{\partial z}-\frac{p\tau_{0}}{A}-\rho_{m}g\sin\theta$$

where $w_{m}$ is the pore size, m; *p* is the pressure in the porous medium, Pa. θ is the angle between the direction of fluid flow and the direction of gravity, in radians.

The oil–water capillary force equation can be expressed by Equation (4).

(4) $$\frac{dQ}{dz}=\frac{\partial }{\partial t}\left[\rho_{m}A\left(U_{m}+\frac{w_{m}^{2}}{2}\right)\right]+\frac{\partial }{\partial z}\left[\rho_{m}w_{m}A\left(U_{m}+\frac{w_{m}^{2}}{2}\right)\right]+\rho_{m}w_{m}Ag\sin\theta +\rho_{m}w_{m}A\frac{\partial \left(pv_{m}\right)}{\partial z}$$

where *v_m_* is the fluid mass velocity, kg·m/s.

Once the oil is removed from the rock surface, it combines with water to create an emulsion due to the tension between the two substances. This emulsion is then carried through the porous medium. The model was established based on the following assumptions, taking into account the intricate nature of fluid movement in the porous medium:

- Fluid flow does not involve heat transfer;
- The viscosity of the oil and water remains constant during the flow;
- The rock surface is required to not adsorb the fluid;
- The effect of the surfactant structure on the interfacial tension between oil and water is not taken into account.

**Figure 13:**
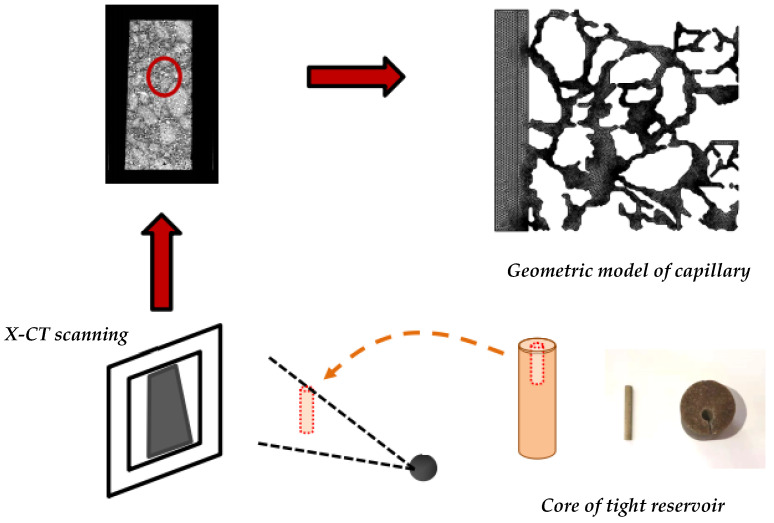
Pore morphology model of porous medium.

The porous structural geometry of the tight reservoir was modeled by performing CT scanning of core sections and mapping pore morphological features. The model constructing process can be demonstrated in Figure 13.

The aforementioned model served as the basis for doing research on the free motion theory of fluids within the porous medium of the tight reservoir. Through the examination of the oil phase saturation and the average liquid phase flow rate, respectively, the flow properties of the oil and water phases within the porous medium were examined. Under varying interfacial tensions, the laws of variation for oil phase saturation and mean flow rate were examined.

### 3.2. Experiment

#### 3.2.1. Materials and Reagents

The materials utilized in this study include deionized water, surfactants, and 40–70 mesh quartz sand. Crude oil was supplied by Daqing Oilfield’s No. 5 Oil Production Plant. The surfactants include 45% sodium diethylhexyl sulfosuccinate (Penetrant-OT, anionic), 39% 2-bis(2-hydroxyethyl)amino]ethyl oleate (2-EO, anionic), 45% dodecyl dimethyl betaine (BS-12, amphoteric), 90% sodium dodecyl benzene sulfonate (SDBS, anionic), 45% sodium dodecyl diphenil ether disulfonate (DB-45, anionic), 60% sodium dodecyl sulfate (SDS, anionic), 80% polysorbate (T-80, nonionic), 35% cocoamidopropyl betaine (CAPB, amphoteric), 70% ethoxylated alkyl sodium sulfate (AES, anionic), 37% lauramidopropyl hydroxysulfobetaine (LHS, amphoteric), 75% sodium lauryl ether sulfate (SLES, anionic), 65% potassium laurate (LPS, anionic), 55% lauramidopropyl betaine (LAPB, amphoteric), and 60% secondary alkane sulphonate sodium (SAS, anionic) produced by the Qingdao USOLF company, China. The model TX-500D rotating drop interfacial tension meter and model A801S dynamic and static contact angle meter, Kino, Boston, MA, USA, were used to measure the interfacial tension and wetting angle, respectively.

#### 3.2.2. Oil-Stripping Efficiency Experiment

The infiltration of external fluids into tight reservoirs is facilitated by the prolonged adsorption of crude oil in the reservoir onto the rock surface. Surfactant solutions that enter the porous medium have the ability to strip oil from the surface of rock particles. During typical displacement testing, it is not possible to regulate the uniformity of the cores across different experiments. Additionally, the test procedure might lead to the emulsification of the surfactant solution with the crude oil, leading to imprecise measurement outcomes. Therefore, several surfactants were employed to extract crude oil from the surface of quartz sand particles, and the factors influencing the effectiveness of crude oil removal were elucidated.

The experimental steps are as follows:

(1) Quartz sand was combined in a 1:6 ratios with crude oil and aged for 24 h at 60 °C;
(2) Deionized water was combined with the surfactant solution, which had a mass concentration of 0.2%;
(3) The centrifuge tube was filled with the oil sand (*m*_1_ = 15 g), and the surfactant was added at a mass ratio of 1:2 to the oil sand. The samples were placed in a centrifuge, which compressed the oil sand and produced the effect of a static stripping test;
(4) Once the oil sand was filtered through the centrifuge tube in Step 3, it was encased and desiccated for a duration of 24 h in an oven;
(5) In step 4, the oil sand was weighed, and Equation (5) was used to determine the oil-stripping efficiency.

(5) $$\eta =\frac{m_{1}-(m_{2}-m_{0})}{m_{1}\times p}$$

where *η* is the oil-stripping efficiency, %, *m*_0_ is the mass of the centrifuge tube, g, and *p* is the oil content, %($m_{1}\times p=2.14g$).

## 4. Conclusions

This research examines the free motion of fluid through a porous medium during spontaneous imbibition, as well as the process of stripping crude oil using a surfactant solution. Upon analysis, it was seen that during the process of stripping, the crude oil underwent progressive aggregation, forming oil droplets that were carried via the porous medium. The oil droplets transferred to the porous medium from the water phase, causing a fluctuating reduction in the oil concentration in the porous medium. Decreased interfacial tension resulted in a reduced rate of spontaneous imbibition in the system. The viscosity of crude oil had a significant impact on the proportion of the oil phase, demonstrating a variable decrease. Lower oil viscosity resulted in an increased flow capacity and a higher average flow rate of the fluid in the system. The oil-stripping efficiency of various surfactants exhibited significant variation, with certain surfactants forming micelles in solution at concentrations over a specific threshold, hence impacting the effectiveness of oil removal. The oil-stripping efficiency had a positive relationship with temperature, as it was influenced by the characteristics of surfactants at elevated temperatures and the alteration in crude oil viscosity. There is a certain set of interfacial characteristics that could be used to improve the efficiency of stripping oil when surfactant solutions are present. Higher oil-stripping efficiencies were obtained when the wetting angle of the rock ranged from 10° to 22° and when the interfacial tension between oil and water was 2 mN/m or less.

## Data Availability

The data presented in this study are available on request from the corresponding author.
